# From Gut to Blood: Redistribution of Zonulin in People Living with HIV †

**DOI:** 10.3390/biomedicines12102316

**Published:** 2024-10-11

**Authors:** Max Augustin, Carola Horn, Meryem Seda Ercanoglu, Vincent Bondet, Ute Sandaradura de Silva, Isabelle Suarez, Seung-Hun Chon, Dirk Nierhoff, Alexander Zoufaly, Christoph Wenisch, Elena Knops, Eva Heger, Florian Klein, Darragh Duffy, Michaela Müller-Trutwin, Clara Lehmann

**Affiliations:** 1 Department I of Internal Medicine, Medical Faculty and University Hospital Cologne, University of Cologne, 50937 Cologne, Germany; carola.horn@uk-koeln.de (C.H.); ute.sandaradura-de-silva@uk-koeln.de (U.S.d.S.); isabelle.suarez@uk-koeln.de (I.S.); 2 Center for Molecular Medicine Cologne (CMMC), 50937 Cologne, Germany; 3 German Center for Infection Research (DZIF), 50937 Cologne, Germany; 4 Department IV of Internal Medicine, Klinik Favoriten, Vienna Healthcare Group, 1100 Vienna, Austria; alexander.zoufaly@gesundheitsverbund.at (A.Z.); christoph.wenisch@gesundheitsverbund.at (C.W.); 5 Faculty of Medicine, Sigmund Freud University, 1020 Vienna, Austria; 6 Institute of Virology, Faculty of Medicine and University Hospital of Cologne, University of Cologne, 50937 Cologne, Germany; meryem.ercanoglu@uk-koeln.de (M.S.E.); elena.knops@uk-koeln.de (E.K.); eva.heger@uk-koeln.de (E.H.); florian.klein@uk-koeln.de (F.K.); 7 Translational Immunology Unit, Institut Pasteur, Université Paris-Cité, 75015 Paris, France; vincent.bondet@pasteur.fr (V.B.); darragh.duffy@pasteur.fr (D.D.); 8 Department of General, Visceral Surgery and Surgical Oncology, University Hospital Cologne, 50937 Cologne, Germany; seung-hun.chon@uk-koeln.de; 9 Department of Gastroenterology and Hepatology, University Hospital of Cologne, 50937 Cologne, Germany; dirk.nierhoff@uk-koeln.de; 10 HIV, Inflammation and Persistence Unit, Institut Pasteur, Université Paris-Cité, 75015 Paris, France; mmuller@pasteur.fr

**Keywords:** zonulin, IFN-α, HIV, chronic immune activation, intestinal barrier, gut-associated lymphatic tissue, GALT, non-infectious comorbidities

## Abstract

Background: Gastrointestinal mucosal damage due to human immunodeficiency virus (HIV) infection leads to microbial translocation and immune activation, contributing to the development of non-infectious comorbidities (NICM) in people living with HIV (PLWH). Additionally, persistent proviral HIV-1 in the gut-associated lymphatic tissue (GALT) can trigger immunological changes in the epithelial environment, impacting the mucosal barrier. However, the role of zonulin, a modulator of epithelial tight junctions in GALT during HIV infection, remains poorly understood. Methods: We measured zonulin in serum and intestinal tissue sections from five treatment-naive (HIV^+^_NAIVE_) and 10 cART-treated (HIV^+^_cART_) HIV^+^ individuals, along with 11 controls (CTRL). We compared zonulin levels with clinical characteristics, inflammatory markers (IFN-α, CXCR3, and PD-1), and the viral reservoir in peripheral blood (PB) and terminal ileum (TI). Results: Upon HIV infection, TI was found to harbor more HIV DNA than PB. Circulating zonulin levels were highest in HIV^+^_NAIVE_ compared to HIV^+^_cART_ or CTRL. Surprisingly, in the gut tissue sections, zonulin levels were higher in CTRL than in HIV^+^ individuals. Elevated circulating zonulin levels were found to be correlated with CD4^+^T-cell depletion in PB and TI, and with intestinal IFN-α. Conclusions: The findings of this study indicate a shift in zonulin levels from the gut to the bloodstream in response to HIV infection. Furthermore, elevated systemic zonulin levels are associated with the depletion of intestinal CD4^+^ T cells and increased gut inflammation, suggesting a potential link between systemic zonulin and intestinal damage. Gaining insight into the regulation of gut tight junctions during HIV infection could offer valuable understanding for preventing NICM in PLWH.

## 1. Introduction

Gastrointestinal (GI) mucosal damage due to human immunodeficiency virus (HIV) infection is a complex phenomenon that has profound implications for the overall health of infected individuals. The disruption of GI barrier integrity results in microbial translocation, which allows gut microbes and their products to enter the systemic circulation, leading to chronic immune activation [1,2]. This, in turn, contributes to the development of various non-infectious comorbidities (NICM) in people living with HIV (PLWH) [3,4], such as cardiovascular disease [5], chronic kidney disease [6], type 2 diabetes mellitus [7] and cancer [8].

Although combined antiretroviral therapy (cART) has significantly improved the overall health outcomes of PLWH, it only partially restores this intestinal damage [9,10]. Therefore, investigating specific biomarkers of the epithelial barrier in the GI tract is crucial for understanding the mechanisms underlying HIV-associated mucosal damage and for developing targeted interventions. Zonulin, for instance, has emerged as a key biomarker in this context. Human zonulin was identified as prehaptoglobin-2 (pre-HP2), previously regarded only as the inactive precursor to haptoglobin-2 (HP2) [11]. Haptoglobin is a hemoglobin-binding protein with immunomodulatory properties, while zonulin is the only known physiological regulator of intercellular tight junctions. It plays a critical role in the trafficking of macromolecules and maintaining the balance between immune tolerance and response [12]. Zonulin has also been implicated in intestinal innate immunity and is upregulated in several autoimmune diseases, including coeliac disease and type 1 diabetes [13]. Finally, zonulin modulates epithelial tight junctions, which are vital for preserving the integrity of the GI barrier [11]. Despite its importance, the role of zonulin in gut-associated lymphatic tissue (GALT) during HIV infection has not been elucidated.

Studies investigating zonulin levels during HIV infections have yielded conflicting results. Some studies have reported an elevation in circulating zonulin levels [14,15,16], suggesting a disruption of epithelial barrier function, while others have observed a decrease in zonulin levels [17], the implications of which are not yet fully understood. Gaining insights into the regulation of gut tight junctions during HIV infection could significantly advance our understanding and contribute to closing the knowledge gap in medical science, particularly by informing strategies for the prevention of NIVM in PLWH.

To address this knowledge gap, we conducted a comprehensive study to investigate zonulin levels in both serum and intestinal tissue sections, aiming to correlate these measurements with inflammatory markers and the virus reservoir in the blood (PB) and terminal ileum (TI) of three distinct groups: cART-treated (HIV^+^_cART_) and treatment-naive (HIV^+^_NAIVE_) HIV-positive patients (HIV^+^) as well as healthy controls (CTRL).

## 2. Materials and Methods

### 2.1. Study Design

In this cross-sectional cohort study, we enrolled 26 patients who attended the outpatient infectious diseases (ID) clinic at the University Hospital Cologne (UHC) between 1 April and 1 November 2018. The study was approved by the Institutional Review Board of the University of Cologne, Germany (16-540). After informed consent, 23 mL (milliliters) of PB, as well as six to ten ileal biopsies from colonoscopy, were obtained from 10 cART-treated (HIV^+^_cART_) and 5 treatment-naïve (HIV^+^_NAIVE_) HIV+ patients, and 11 healthy individuals (CTRL). Colonoscopies were performed at the Department of Endoscopy of the UHC. The inclusion criteria were: 18–65 years of age and infection with HIV-1. Exclusion criteria included contraindications for sedation or endoscopy and inflammatory bowel diseases. The patients’ clinical characteristics and coinfections at the time of biopsy and blood draws were documented (Table 1).

### 2.2. Cell Isolation

Peripheral blood mononuclear cells (PBMCs) and lamina propria mononuclear cells (LPMNCs) were separated using density centrifugation on a Ficoll gradient (L 6115, Biochrom, Cambridge, UK), as described by Lehmann et al. [18], and were kept frozen at −180 °C in liquid nitrogen for future analysis. The ileal biopsies from colonoscopy were immediately placed in 5 mL tissue culture medium (RPMI 1640, F 1215, Biochrom, Cambridge, UK), augmented with 10% penicillin/streptomycin and 25 µg/mL amphotericin B, used as supernatant after removal of biopsies. An extra-ileal biopsy was embedded in paraffin and stored for immunohistochemical analysis.

### 2.3. Quantification of Zonulin in Serum and Gut Tissue Sections

In the serum, circulating zonulin levels (ng/mL) were measured by enzyme-linked immunosorbent assay (ELISA) according to the manufacturer’s instructions (Immundiagnostik, Bensheim, Germany). Intestinal zonulin expression was assessed in the gut sections of the terminal ileum using semi-quantitative immunohistochemistry. Fresh tissues were fixed in 4% formalin and embedded in paraffin. Sections (3 μm) were stained with hematoxylin and eosin. Immunohistochemistry (IHC) was performed using the anti-human haptoglobin precursor LS-B32 (Lifespan Biosciences, Lynnwood, WA, USA) and the appropriate secondary antibody (AB-6720, Abcam, Cambridge, UK). Using a 4×, 20× and 40× objective, photos of the sections were taken using a BZ-9000 microscope (Keyence, Osaka, Japan) with a 20× magnification numerical aperture objective lens (Nikon, Tokyo, Japan). Subsequently, intestinal zonulin expression was repeatedly scored (3 times) in an area of total 1.2 mm^2^ in each patient. Medians (interquartile range (IQR)) of the expression of intestinal zonulin were calculated per patient and were subsequently compared as described in the statistical analysis section.

### 2.4. Flow-Cytometry and FACS Sorting

Thawed mononuclear cells were stained with CD3-APC-H7 (1:40, 560176, Becton Dickinson (BD), Franklin Lakes, NJ, USA), CD4-FITC (1:40, 555346, BD, Franklin Lakes, NJ, USA), and CD45RO APC (1:10, 130-109-430, Miltenyi Biotec, Bergisch Gladbach, Germany). The expression of CXCR3 BV510 (1:20, 353726, Biolegend, San Diego, CA, USA) and PD-1 BV421 (1:20, 329920; Biolegend, San Diego, CA, USA) was assessed by geometric mean fluorescence intensities (gMFI). Exemplary fluorescence-activated cell sorting (FACS) plots were obtained in our previous work [19,20]. Samples were sorted on a BD FACSAria Fusion flow cytometer (Franklin Lakes, NJ, USA) into 2 mL collection tubes at 4-way purity and incubated without prior culturing with the following antibodies. Isolation did not affect the detection of membrane proteins using antibodies. Flow cytometry was performed on a BD FACSAria Fusion flow cytometer (Franklin Lakes, NJ, USA).

### 2.5. DNA Extraction and HIV-1 DNA Quantification

Total human immunodeficiency virus 1 (HIV-1) deoxyribonucleic acid (DNA) was extracted from sorted memory CD4^+^ T cells using the QIAamp DNA Blood Kit (51104; Qiagen, Hilden, Germany), according to the manufacturer’s instructions. HIV-1 copies of the eluate were quantified using the Versant HIV-1 ribonucleic acid (RNA) 1.5 Assay (kPCR) from Siemens (Munich, Germany), with a range of quantification of 37 to 11 × 10^6^ copies/mL. Cell-associated HIV-1 DNA copies/mL was normalized to β-globin levels and expressed as HIV-1 DNA copies per PBMC × 10^6^.

### 2.6. Ultrasensitive Quantification of Interferon Alpha (IFN-α) by SIMOA

Ultrasensitive digital ELISA (Simoa; Quanterix, Billerica, MA, USA; femtograms (fg)/mL) was performed on thawed serum and gut tissue supernatants to quantify interferon alpha subtype 1(IFN-α) levels as previously described [21]. Gut biopsies remained for an average of 4 h in tissue culture medium and were later used for ultrasensitive IFN-α quantification.

**Table 1:** Clinical characteristics of cART-treated and treatment-naive HIV positive individuals (HIV^+^) and healthy controls (CTRL).

Patients	*n*, [Years]	Age, [Years]	HIV-1, [22]	cART, [Years]	CD4^+^, [Cells/mm^3^]	CD4^+^, [%]	CD4^+^ Nadir, [Cells/mm^3^]	Plasma Viral Load, [RNA Copies/mL]	Aviremia, [Years]
HIV^+^_cART_	10 _(8♂, 2♀)_	57 _(IQR 49–63)_	14 _(IQR 8–20)_	12 _(IQR 8–13)_	561_(IQR 390–860)_	32 _(IQR 24–34)_	192 _(IQR 57–423)_	40 _(IQR 40–40)_	9 _(IQR 5–11)_
HIV^+^_NAIVE_	5 _(5♂, 0♀)_	48 _(IQR 39–51)_	5 _(IQR 2–19)_	0	70_(IQR 30–405)_	16 _(IQR 3–24)_	40 _(IQR 30–380)_	45k _(13k–15.7M)_	0
CTRL	11 _(5♂, 6♀)_	59 _(IQR 52–65)_	n.a.	n.a.	n.a.	n.a.	n.a.	n.a.	n.a.

cART, combined antiretroviral therapy; HIV, human immunodeficiency virus; HIV^+^, HIV positive individuals; HIV^+^_cART_, cART-treaded HIV^+^; HIV^+^_NAIVE_, treatment-naive HIV^+^; *n*, number; CD4^+^, CD4 positive T cells; RNA, ribonucleic acid; mL, milliliters; mm^3^, cubic millimeters; IQR, interquartile range; k, thousand; M, million; n.a., not applicable; ♂, male; ♀, female.

### 2.7. Statistical Analysis

Statistical analyses were conducted using GraphPad Prism Software Version 9.3.1. (GraphPad Software, La Jolla, CA, USA). Normality was assessed using the Kolmogorov–Smirnov or Shapiro–Wilk tests. *p* values of 0.05 and lower was set at *p* < 0.05. Significant differences in non-parametric distributions (*p* < 0.05) were tested using Kruskal–Wallis tests with Dunn’s multiple comparisons, two-tailed Mann–Whitney tests, or Wilcoxon matched-pairs tests, as applicable. Spearman’s r was used to describe non-parametric correlations. Correlation analyses were performed only for the HIV^+^_cART_ group. The statistical parameters (value of *n*, statistical calculation, etc.) are stated in the figure legend. If not otherwise stated, all variables were represented as medians with interquartile ranges (IQR).

## 3. Results

### 3.1. Patient Characteristics

The median ages of the 10 cART-treated HIV-positive patients (HIV^+^_cART_) and treatment-naive HIV-positive patients (HIV^+^_NAIVE_) were 57 years (IQR 49–63 years) and 48 years (IQR 39–51 years), respectively (Table 1). HIV^+^_cART_ had a CD4 cell count of 561 (IQR 390–860) per microliter (µL) and a plasma viral load below 40 HIV-1 RNA copies per milliliter (ml). In contrast, HIV^+^_NAIVE_ presented with a CD4+ cell count of 70/µL (IQR 30–405) and a plasma HIV-1 viral load of 44,600 (IQR 13,280–15,700,000) RNA copies/mL (Table 1). Coinfections were present in two chronic HIV patients treated on cART.

### 3.2. Diminished Intestinal Zonulin Expression in the Terminal Ileum of HIV-Positive Patients Compared to Healthy Controls

**Figure 1:**
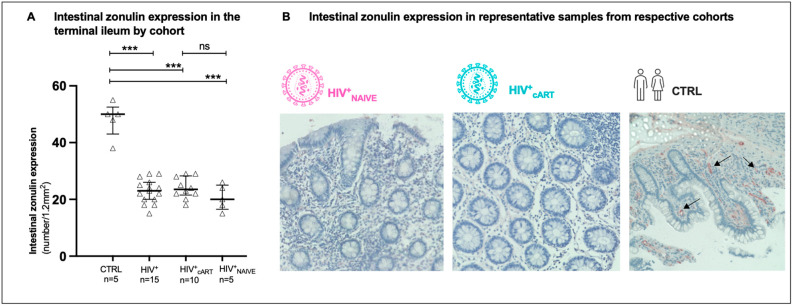
Analysis and detection of intestinal zonulin expression in gut tissue sections of the terminal ileum. (**A**) Median (IQR) intestinal zonulin expressing cells (number/1.2 mm^2^) by cohort. Results from semiquantitative immunohistochemical analysis. (**B**) IHC detection of intestinal zonulin (black arrows) in representative samples from one treatment-naive (HIV^+^_NAIVE_) and one cART-treated (HIV^+^_cART_) HIV-positive individual and one healthy control (CTRL) are shown. Original magnification: ×40. Data information: Variables are represented as medians with interquartile ranges (IQR). For statistical analysis, normality was assessed using the Shapiro–Wilk or Kolmogorov–Smirnov test. For non-parametric distributions, Kruskal–Wallis tests with Dunn’s multiple comparisons were used to test for statistical significance. *** *p* ≤ 0.001, statistically significant; ns, not significant. *n*, number; mm^2^, square millimeter.

As zonulin drives the regulation of tight junctions of the epithelial gut barrier in the terminal ileum (TI), median zonulin expression was assessed by semiquantitative, immunohistochemical analysis in gut tissue sections of our cohorts (number/1.2 mm^2^) (Figure 1). The TI of HIV-negative controls expressed significantly more median intestinal zonulin when compared to HIV^+^ controls (CTRL: 50.0 (IQR 43–52.5, HIV^+^: 23 (IQR 20–26), *p* = 0.0001, Figure 1A). In addition, median intestinal zonulin expression declined from cART-treated (HIV+_cART_: 23.5 (IQR 21.5–28.3)) to treatment-naive HIV-positive patients (HIV^+^_NAIVE_: 20 (IQR 16.5–25)). However, no significant differences were detected (*p* = 0.2181; Figure 1A).

### 3.3. Treatment-Naive HIV^+^ Patients Exhibit the Highest Circulating Zonulin Levels

**Figure 2:**
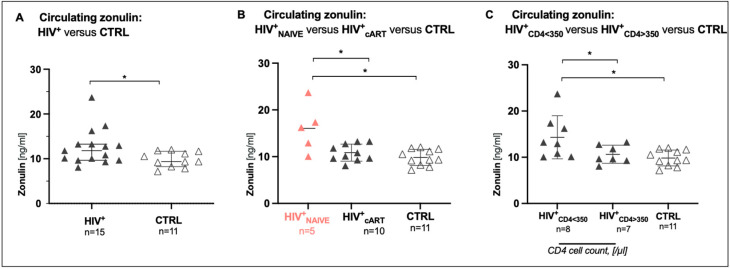
Circulating zonulin by cohort. Circulating zonulin levels (ng/mL) were higher (**A**) in HIV-positive patients (HIV^+^) than in controls (CTRL), (**B**) in treatment-naive HIV^+^ (HIV^+^_NAIVE_) than in cART-treated HIV^+^ (HIV^+^_cART_, *p* = 0.04) (HIV^+^_NAIVE_ > HIV^+^_cART_ > CTRL), and (**C**) in patients with low (HIV^+^_<350_) versus high (HIV^+^_>350_) CD4 cell count. Data information: Variables are represented as medians with interquartile ranges (IQR). For statistical analysis, normality was assessed using the Shapiro–Wilk or Kolmogorov–Smirnov test. For non-parametric distributions, Kruskal–Wallis tests with Dunn’s multiple comparisons were used to test for statistical significance. *p* < 0.05 indicated statistical significance: * *p*  ≤  0.05. *n*, number; ng, nanogram; mL, millimeter; ≤, less or equal.

To address the potential relocation of intestinal zonulin to peripheral blood (PB), circulating zonulin levels were measured in the serum of our cohorts. In contrast to gradually declining epithelial zonulin levels in the gut from CTRL to HIV^+^_cART_ and particularly to HIV^+^_NAIVE_, in serum, zonulin levels (ng/mL) were significantly higher in HIV^+^ patients than in healthy controls (Figure 2A). Moreover, circulating zonulin levels were highest in HIV^+^_NAIVE_ when compared to HIV^+^_cART_ (HIV^+^_NAIVE_: 16.2 (IQR 11.5–20.5), HIV^+^_cART_: 10.5 (IQR 9.6–12.9), *p* = 0.04), and CTRL (CTRL: 9.4 (IQR 8.2–11.7, *p* = 0.0087) (Figure 2B, HIV^+^_NAIVE_ > HIV+_cART_ > CTRL). To better portray the immune function in HIV^+^ patients, zonulin levels were stratified by CD4 cell counts lower (HIV^+^_<350_, *n* = 8) and higher (HIV^+^_>350_, *n* = 7) than 350 cells/µL. Here, zonulin levels were highest in HIV^+^_<350_: 13.1 (IQR 10.3–17.1) when compared to HIV^+^_>350_: 9.7 (IQR 9.3–12.8), *p* = 0.05) and CTRL (CTRL: 9.4 (IQR 8.2–11.7), *p* = 0.0121) (Figure 2C, HIV^+^_<350_ > HIV+_>350_ = CTRL).

### 3.4. Terminal Ileum: A Major Reservoir of HIV-1 DNA

**Figure 3:**
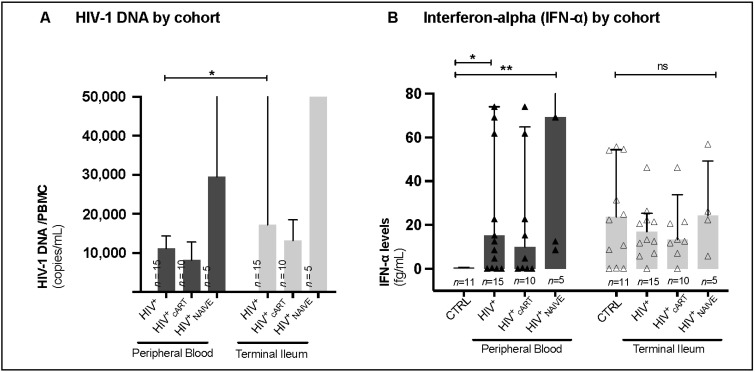
Human immunodeficiency virus (HIV-1) DNA and interferon-alpha (IFN-α) by cohort. (**A**) HIV-1 gut tissue reservoir was measured in proviral HIV-1 DNA (copies/mL) and (**B**) interferon-alpha levels (fg/mL) by site and cohort. Data information: Variables are represented as medians with interquartile ranges (IQR). For statistical analysis, normality was assessed using the Shapiro–Wilk or Kolmogorov–Smirnov test. For non-parametric distributions, Kruskal–Wallis tests with Dunn’s multiple comparisons were used to test for statistical significance. *p* < 0.05 indicated statistical significance: * *p* ≤ 0.05, ** *p* ≤ 0.01; and ns, not significant. HIV, human immunodeficiency virus; cART, combined antiretroviral therapy; HIV^+^, HIV-positive individuals; HIV^+^_cART_, cART-treated HIV^+^; HIV+_NAIVE_, treatment-naive HIV^+^; *n*, number; mL, milliliter; fg, femtogram; ≤, less or equal.

To address the role of circulating and intestinal zonulin in HIV-1 viral tissue reservoirs and chronic immune activation, proviral HIV-1 DNA (viral copies/PBMC × 10^6^) and interferon-α (IFN-α, fg/mL) were measured in the peripheral blood (PB) and terminal ileum (TI) of our cohorts. While the TI of HIV+ patients harbored significantly more HIV-1 DNA than PB (HIV-1 DNA copies/PBMC × 10^6^; PB: 11,140 (IQR 4240–14,353), TI: 17,200 (IQR 13,280–151,700), *p* = 0.0264, Figure 3A), IFN-α levels in our HIV^+^ cohorts were distributed similarly between PB and TI (fg/mL; PB: 15.3 (IQR 0.4–74.0), TI: 16.9 (IQR 8.2–25.3), *p* > 0.05, Figure 3B).

In PB, significant increases were only detected in IFN-α levels between HIV^+^ and HIV^+^_NAIVE_ when compared with CTRL (fg/mL; HIV^+^: 15.3 (IQR 0.4–74.0), HIV^+^_cART_: 10.0 (IQR 0.2–64.9), HIV^+^_NAIVE_: 69.2 (IQR 10.6–7228), CTRL: 0.8 (IQR 0.1–0.9), *p* = 0.0137 and *p* = 0.0012, Figure 3B). In contrast, IFN-a levels in TI were distributed similarly (*p* > 0.05) between cohorts (fg/mL; HIV^+^: 20.6 (IQR 9.4–36.2), HIV^+^_cART_: 16.7 (IQR 10.6–27.7), HIV^+^_NAIVE_: 24.3 (IQR 9.9–49.2), CTRL: 31.4 (IQR 10.7–55.7), Figure 3B). In comparison to treatment-naive individuals, IFN-α levels in the cART-treated cohort were reduced by 90% in PB and only 30% in TI.

### 3.5. Correlations between Increased Circulating Zonulin, Intestinal CD4+ T-cell Depletion, and Increased Inflammation in the Terminal Ileum

**Figure 4:**
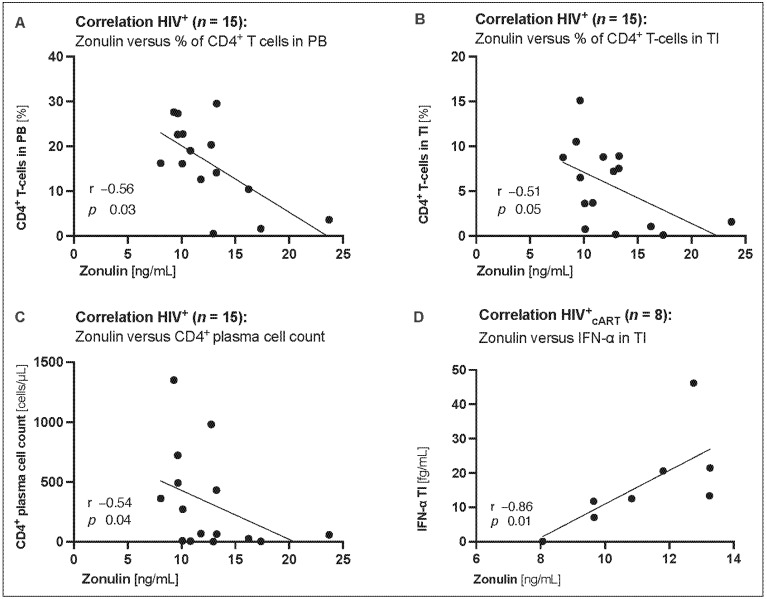
Correlations of circulating zonulin with clinical characteristics and markers of HIV disease progression and chronic immune activation. Circulating zonulin levels (ng/mL) were negatively correlated with CD4 positive (CD4^+^) T-cell frequencies in (**A**) the peripheral blood (PB) (r = −0.56, *p* = 0.03) and (**B**) terminal ileum (TI) (r = −0.54, *p* = 0.04), and with (**C**) CD4+ plasma cell count (r = −0.51, *p* = 0.05) of HIV^+^ and (**D**) positively correlated with interferon-alpha (IFN-α) (r = 0.86, *p* = 0.01) of HIV^+^_cART_ in the TI. Data information: Variables are represented as medians with interquartile ranges (IQR). For statistical analysis, normality was assessed using the Shapiro–Wilk or Kolmogorov–Smirnov test. Spearman’s r was used to describe non-parametric correlations. Correlation analyses for (**D**) were performed for HIV^+^_cART_ only. *p* < 0.05 indicated statistical significance. HIV, human immunodeficiency virus; *n*, number; ng, nanogram; mL, milliliters; µl, microliters.

Finally, to assess the role of zonulin in immune function and chronic immune activation, circulating zonulin levels were correlated with clinical characteristics, as well as activation and exhaustion markers such as CD4^+^ T-cell frequencies, IFN-α, CXCR3, and PD-1 expression. Circulating zonulin levels were negatively correlated with CD4^+^ T-cell frequencies in both the PB (r = −0.56, *p* = 0.03, Figure 4A) and TI (r = −0.51, *p* = 0.05, Figure 4B), as well as with the CD4 plasma cell count in PB (r = −0.54, *p* = 0.04, Figure 4C). Moreover, serum zonulin levels showed a positive correlation with IFN-α levels in the terminal ileum (r = 0.86, *p* = 0.01, Figure 4D). However, no significant correlations were found between zonulin and CD4^+^ memory T cell frequencies, plasma viral load, proviral HIV-1 reservoir, CXCR3, or PD-1 expression.

## 4. Discussion

The successful daily distribution of combined antiretroviral therapy (cART) plays a pivotal role in achieving and maintaining undetectable HIV-1 viremia in the peripheral blood of PLWH, rendering HIV untransmittable from one individual to another (U = U status) [23]. However, despite the remarkable effectiveness of cART in controlling viral replication, HIV-1 reservoirs persist in gut-associated lymphatic tissue (GALT) and are already established during primary HIV infection. Consistent with previous findings in macaques [24,25] and humans [19,20,26], our study demonstrated that the terminal ileum (TI) serves as the largest proviral HIV-1 reservoir, even in the presence of cART (Figure 3A). Notably, despite cART, persistent proviral HIV-1 in the GALT might contribute to immunological changes in the epithelial microenvironment, interfering with the mucosal intestinal barrier [27]. To explore this further, we assessed intestinal integrity in cART-treated HIV^+^ (HIV^+^_cART_), treatment-naïve HIV^+^ (HIV^+^_NAIVE_), and healthy controls (CTRL) using regulatory proteins of intestinal tight junctions and gut fat metabolism, specifically zonulin [11] and intestinal fatty acid-binding protein (I-FABP) [28], respectively. Intriguingly, we observed that the number of zonulin-expressing cells (per 1.2 mm^2^) decreased in the TI upon HIV infection, a first-time observation (Figure 1), while in line with previous data [14,15,16], circulating zonulin levels increased in the PB of HIV^+^ individuals compared to CTRL, with the highest levels in HIV^+^_NAIVE_ > HIV^+^_cART_ = CTRL (Figure 2A,B). These findings suggest redistribution of zonulin from the gut to the blood in response to HIV infection. Elevated levels of zonulin circulating in the blood have been observed as indicators of impaired intestinal integrity in various other conditions such as autoinflammatory [29], autoimmune [13] and coeliac diseases [11].

Interestingly, conflicting results regarding circulating zonulin levels in PLWH have been reported, with some studies showing increased levels [14,15,16] and others demonstrating decreased levels [17]. Whether increased or decreased, circulating zonulin levels may reflect the qualitative function of an individual’s immune system. Thus, systemic zonulin levels can predict mortality in cART-treated HIV-positive patients with a history of acquired immune deficiency syndrome (AIDS) [17]. Moreover, circulating zonulin levels could distinguish treatment-naive from cART-treated individuals with 87.7% sensitivity and 69% specificity [15]. In contrast to the redistribution of zonulin from the gut to the blood upon HIV infection, I-FABP (Figure S1) was equally distributed in HIV-positive and -negative patients in this study, and its expression was therefore not assessed in the gut sections of the terminal ileum.

The well-described compromised gut epithelial barrier integrity in PLWH [2,30] highlights GALT HIV-1 reservoirs as a potential critical anatomical sanctuary, where persistent and ongoing chronic immune activation influences the increased occurrence of non-infectious comorbidities (NICM) [3,4]. This led us to expect higher IFN-α levels in the TI than in the PB. However, our results revealed that interferon-alpha levels (fg/mL), a measure of chronic immune activation, were similarly distributed between the PB and TI of cART-treated HIV-positive patients (Figure 3B). However, after initiation of cART, zonulin levels were lowered by 90% in the PB and only 30% in the TI (Figure 3B). This suggests that cART reduced immune activation in the PB, whereas the TI remained more susceptible to chronic immune activation. In this study, detailed analyses were conducted to explore the interplay between zonulin levels and individual immune function and capacity. Zonulin levels were the highest in HIV+ patients with the lowest CD4^+^ plasma cell count (HIV^+^_<350_ > HIV+_>350_ > CTRL, Figure 2C). In addition, elevated plasma zonulin levels showed significant correlations with depletion of CD4+ T cells and CD4^+^ plasma cell counts in PB and TI (Figure 4A–C), as well as with chronic immune activation measured by gut interferon-alpha levels (Figure 4D). However, no significant correlations were found between zonulin and CD4^+^ memory T cell frequencies, plasma viral load, proviral HIV-1 reservoir, or the activation and exhaustion markers CXCR3 and PD-1, respectively. In the present study, circulating zonulin, and not I-FABP, mirrored impaired intestinal integrity in HIV-positive patients. Circulating zonulin levels could be used as an indicator of gut epithelial barrier dysfunction in PLWH. However, in this study, I-FABP levels did not show significant differences between HIV-positive and HIV-negative patients, suggesting its limited utility as a biomarker in this context.

Although cART significantly normalized zonulin, IFN-α and HIV DNA levels in the PB, it failed to achieve that in the TI, suggesting that GALT may serve as an ongoing anatomic sanctuary for impaired intestinal integrity, chronic immune activation, and HIV-1 proviral persistence. These factors may collectively fuel the pathogenesis of noninfectious comorbidities in PLWH, even in the presence of effective cART.

While our study offers valuable insights, it has several limitations. First, it should be noted that the small sample sizes of predominantly male patients in our study limited the statistical power. Second, the study required colonoscopies for data collection, resulting in a limited number of available biopsies and consequently, a restricted number of cells for analysis. Third, while the analysis of circulating zonulin was quantitative (ng/mL), the expression of intestinal zonulin was scored in a semiquantitative way. Fourth, due to the complexities involved in acquiring gut biopsies, this study was conducted using a cross-sectional design. However, a longitudinal study design would have been more suitable for assessing intra-individual outcomes over time and gaining deeper insight into the dynamics of the parameters under investigation. Fifth, measuring total proviral HIV DNA in cell lysates as a surrogate for HIV-1 tissue reservoirs may not fully represent the entire reservoir population and may have no information value with regard to the replication capacity.

## 5. Conclusions

In conclusion, the data on circulating and intestinal zonulin suggest that following HIV infection, zonulin levels decrease in the gut, but increase in plasma. This redistribution is associated with the loss of intestinal CD4^+^ T cells and heightened gut inflammation, suggesting that elevated levels of systemic zonulin correlate with disease progression and intestinal damage. Notably, only the redistribution of zonulin, rather than I-FABP, was indicative of gut epithelial intestinal integrity during HIV infection. Understanding the regulation of gut tight junctions in the context of HIV infection may be crucial for understanding the emergence of noninfectious comorbidities in PLWH and could open new directions for novel therapeutic approaches.

## Data Availability

Raw data were generated at the Medical Faculty and University Hospital Center. The data supporting the findings of this study are available from the corresponding author (C.L.) on request.

## Supplementary Materials

The following supporting information can be downloaded at: https://www.mdpi.com/article/10.3390/biomedicines12102316/s1, Figure S1: Intestinal fatty acid binding protein (I-FABP) by cohort.
