# Supplementary figures and images for: From Gut to Blood: Redistribution of Zonulin in People Living with HIV
Source: Biomedicines. 2024 Oct 11;12(10):2316. doi: 10.3390/biomedicines12102316 (PMC11505231; doi:10.3390/biomedicines12102316)

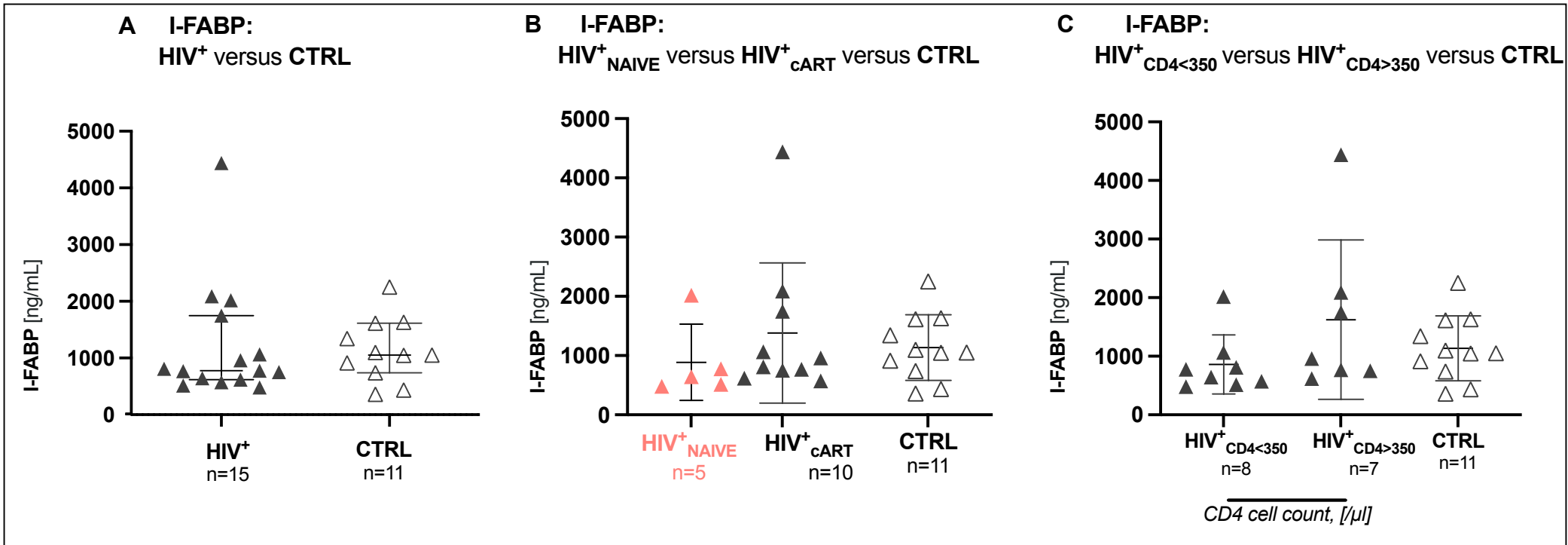

**Figure S1** Intestinal fatty acid binding protein (I-FABP) by cohort.

Supplement: Supplementary file 1 [file biomedicines-12-02316-s001.zip › biomedicines-3198704-supplementary.pdf]
